# Visceral leishmaniasis in kidney transplant recipients and candidates: an integrative review of the last 20 years

**DOI:** 10.1590/2175-8239-JBN-2024-0138en

**Published:** 2025-05-19

**Authors:** Osvaldo Mariano Viana, Ednaldo Pereira Lima, Beatriz Fontenele Felix, Gustavo Ferreira da Silva, Pedro Yago Lima de Mesquita, Francisco Mikael Alves Mota, Evelyne Santana Girão, Maria Alice Sperto Ferreira Baptista, Celia Sebastiana de Jesus Fazzio, Elizabeth De Francesco Daher, Wanessa Trindade Clemente

**Affiliations:** 1 Universidade Federal do Ceará, Faculdade de Medicina, Departamento de Medicina Clínica, Fortaleza, CE, Brazil.; 2 Universidade Federal do Ceará, Hospital Universitário Walter Cantídio, Fortaleza, CE, Brazil.; 3 Hospital de Base de São José do Rio Preto, São José do Rio Preto, SP, Brazil.; 4 Universidade Federal de Minas Gerais, Faculdade de Medicina, Departamento de Propedêutica Complementar, Belo Horizonte, MG, Brazil.

**Keywords:** Kidney Transplantation, Leish-maniasis, Visceral, Neglected Diseases, Immunocompromised Host

## Abstract

**Introduction:**

Leishmaniasis is a potential concern for solid organ transplant (SOT) recipients, particularly those from endemic regions. Among SOT procedures, kidney transplantation (KT) is the most common. This study aims to synthesize the evidence about visceral leishmaniasis (VL) in KT candidates and recipients, with a focus on risk factors and associated outcomes.

**Methods:**

This integrative review analyzed studies from the past 20 years, focusing on disease profile, treatment, prognosis, and risk of asymptomatic infection.

**Results:**

A total of 32 articles were included. Of the KT recipients, 85.7% were male, with an average age of 42.5 years. The average timespan since symptom onset was 54.7 months. Renal function impairment was reported in 64% of patients, with an associated mortality rate of 15%. Post-treatment relapse occurred in 10–37.5% of patients. Among KT candidates, 13.9% were seropositive for *Leishmania spp*.

**Conclusion:**

VL is an infrequent condition among KT recipients, limiting the quality of the available evidence. Early detection and prompt treatment are crucial for improving outcomes. While renal function impairment is common, it rarely leads to graft rejection. In the reviewed studies, the coexistence of VL and cutaneous or mucocutaneous forms was linked to higher mortality. Recurrences are common and require individualized management strategies. Hemotransfusion poses a potential infection risk, although routine screening in blood banks is not yet recommended.

## Introduction

Visceral leishmaniasis (VL) is a neglected tropical parasitic disease, caused by a group of intracellular protozoa belonging to the *Leishmania donovani* complex, also known as “kala-azar”. These pathogens, transmitted by the bite of the female *Phlebotomus* or *Lutzomyia* sand fly, have tropism for reticuloendothelial cells, mainly those of the spleen, liver, and bone marrow^1^.

In transplant recipients, the use of immuno-suppressant agents is essential to prevent allograft rejection, which is associated with symptomatic manifestation of the infection through mechanisms that have not been fully elucidated and can reactivate asymptomatic infections^2, 3, 4^. Kidney transplant (KT) recipients may become infected through blood transfusion, allograft transmission, or further exposure to infected sand flies^4^. Information regarding asymptomatic VL in transplant candidates is scarce, and disease recognition in the transplant recipient may therefore be delayed^5^.

The diagnosis involves the identification of parasites mainly by microscopy (of the bone marrow and, less frequently, of the spleen). Culture isolation, rK39 rapid immunochromatographic test (antigen detection), and polymerase chain reaction (PCR) from peripheral blood or bone marrow^6^ can also be used. Serology, through indirect fluorescent antibody test (IFAT), enzyme-linked immunosorbent assay (ELISA), and direct agglutination test (DAT), is an option, although each test may present different levels of sensitivity and specificity^6^. The preferred treatment in these patients requires a systemic approach, and a reduction of the immunosuppression dose is often advisable. The drug of choice is liposomal amphotericin B (L-AmB) and, alternatively, pentavalent antimonials, which can cause kidney damage. Other less common options include miltefosine, pentamidine, and paromomycin^7^.

VL is an uncommon infection in the post-transplant period, even within endemic regions. Among solid organ transplant (SOT) recipients, VL is most frequently reported in KT, accounting for 77% of cases^1^. This finding likely reflects the higher prevalence of this type of transplant among SOT^4, 8^. Typically, VL infection causes fever, pancytopenia, hepatosplenomegaly, and weight loss. However, these are the classic but not universal manifestations of VL. In some cases, the disease can also lead to nephropathy, caused by inflammatory infiltration and glomerular sclerosis, which can lead to graft dysfunction in KT recipients^1, 5, 9^.

This study aimed to elucidate the current scientific knowledge on leishmaniasis infection and outcomes among KT candidates and recipients, emphasizing the adverse effects related to treatment and renal health in this population.

## Methods

An integrative review was conducted to discuss the clinical profile and diagnosis of VL infection in KT candidates and recipients, in addition to outlining treatments and prognostic factors.

The search was conducted using the descriptors “(‘Visceral Leishmaniasis’) AND (‘Kidney Transplant’ OR ‘Renal Transplant’)” in three databases (PubMed, Google Scholar, and MEDLINE) in March 2024. The search covered the period from 2004–2024, selecting only articles in English. Case reports and series, retrospective cohorts, and systematic reviews were all included. Duplicates were manually removed and abstracts from conferences were excluded.

The inclusion criteria were studies describing patients with VL or asymptomatic *Leishmania* spp. infection diagnosed in KT recipients and candidates published in indexed journals. The exclusion criteria were studies focused on non-visceral forms of leishmaniasis (cutaneous and mucocutaneous), those involving non-KT patients or transplant types other than KT (e.g., liver or hematopoietic stem cell transplants), and manuscripts that failed to specify the transplant type.

The article selection process was conducted by two authors (OMVN and PYLM), and any uncertainties regarding inclusion or exclusion were discussed within the group of authors for consensus. The final list of selected articles was documented in a spreadsheet, which was managed by the authors, for subsequent data analysis.

## Results

### Included Studies

**Figure 1:**
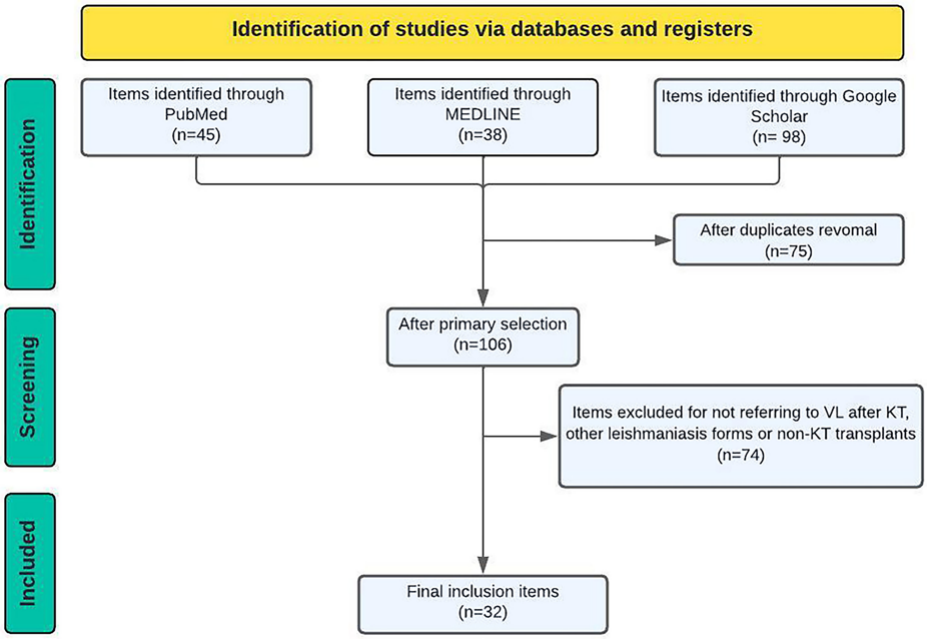
Study selection flowchart. Preferred Reporting Items for Systematic Reviews and Meta-Analyses (PRISMA) flowchart adapted for integrative reviews.

Figure 1 shows a flowchart detailing the selection process of studies included in this review.

The authors categorized the manuscripts into subgroups as follows: case reports or series on KT recipients^9, 10, 11, 12, 13, 14, 15, 16, 17, 18, 19, 20, 21, 22, 23, 24, 25, 26, 27, 28^; observational studies on asymptomatic infection in KT candidates^5, 29, 30, 31, 32, 33^; observational studies on KT patients who developed VL^34, 35, 36, 37, 38^; and a systematic review of VL in KT recipients^1^.

Epidemiology of VL in KT recipients, prevalence of asymptomatic infection in KT candidates, clinical and laboratory data, biopsy findings, therapeutic management, and relapses were observed in each of the included studies.

### Epidemiology

The selection of articles yielded 20 articles from case reports and case series containing data from 25 patients (Table 1). Most reports originate from endemic countries such as those in the Mediterranean Basin, Brazil, India, Saudi Arabia, and Iran^13^. Three patients reported travelling: one to Spain and Tunisia^12^, one to France and Morocco^19^, and one to Brazil and Thailand^25^.

**Table 1:** Data from case reports and small case series (<8 patients) of visceral leishmaniasis among kidney transplant recipients

	Symptoms	Time after KT	SCr	CL/MC presentation	Country	Death	Treatment
Busutti et al.^9^, 2023	Night sweat and fever	18	3.7	None	Italy	No	L-AmB
Rana et al.^10^, 2022	Abdominal pain and weight loss	48	2.5	None	India	No	L-AmB
Marques et al.^11^, 2020	Fever and oral ulcers	108	–	Both	Portugal	Yes	L-AmB
Dettwiler et al.^12^, 2010	Fever, anorexia, weight loss, asthenia, and hepatoesplenomegaly	69	2.71	None	Travel to endemic area	Yes	L-AmB
Bouchekoua et al.^13^, 2014	Fever, anorexia, asthenia, and weight loss	17	4.18	None	Tunisia	No	Glucantime
Kardeh et al.^14^, 2023	Fever, chills, and malaise	23	1.61	None	Iran	No	L-AmB
Madhyastha et al.^15^, 2016	Fever and oral ulcers	62	4.3	MC	India	No	L-AmB
Sánchez et al.^16^, 2018 **(Case 1)**	Fever and adenopathies	24	–	None	Spain	No	L-AmB
Sánchez et al.^16^, 2018 **(Case 2)**	Fever	192	–	None	Spain	No	L-AmB
Simon et al.^17^, 2011 **(Case 1)**	Oral ulcers	120	–	MC	Italy	Yes	L-AmB
Simon et al.^17^, 2011 **(Case 2)**	Fever and general deterioration	204	–	CL	Italy	No	L-AmB*
Zumrutdal et al.^18^, 2010	Fever, hepatosplenomegaly, and weight loss	60	2,2	None	Turkey	No	L-AmB/ Allopurinol*
Duvignaud et al.^19^, 2015	Fever, asthenia, and diarrhea	72	1.54	None	Travel to endemic area	No	L-AmB/Pentamidine
Yücel et al.^20^, 2013	Fever and skin papules	84	–	CL	Turkey	Yes	L-AmB
Pedroso et al.^21^, 2014	Fever, chills, and indisposition	192	–	None	Italy	Yes	L-AmB*
Oliveira et al.^22^, 2008 **(Case 1)**	Fever, asthenia, anorexia, and diarrhea	5	1.8	None	Brazil	No	L-AmB
Oliveira et al.^22^, 2008 **(Case 2)**	Fever, chills, anorexia, and weight loss	36	2.5	None	Brazil	No	L-AmB
Oliveira et al.^22^, 2008 **(Case 3)**	Fever, anorexia, asthenia, lymphadenopathy, and hepatosplenomegaly	36	1.2	None	Brazil	No	L-AmB
Oliveira et al.^22^, 2008 **(Case 4)**	Diarrhea, weight loss, abdominal pain, asthenia, anorexia, and fever	8	2.7	None	Brazil	No	L-AmB
Rancan et al.^23^, 2022	Diarrhea, fever, and hepatosplenomegaly	48	–	None	Brazil	Yes	L-AmB
Jha et al.^24^, 2012	Fever, cervical lymphadenopathy, and splenomegaly	84	1.1	MC	Nepal	No	L-AmB
Pêgo et al.^25^, 2013	Fever, indisposition, weakness, night sweat, and cachexia	17	1.48	None	Travel to endemic area	No	L-AmB
Prasad et al.^26^, 2011	Fever, asthenia, and myalgia	84	5.2	None	India	No	L-AmB
Gembillo et al.^27^, 2021	Anemia, fever, and urinary retention	36	4	None	Italy	No	L-AmB
Keitel et al.^28^, 2018	Fever, myalgia, weight loss, weakness, and splenomegaly	33	–	None	Brazil	No	L-AmB

Abbreviations – KT: kidney transplant; SCr: serum creatinine (mg/dL) at diagnosis; CL/MC: presence of cutaneous (CL); mucocutaneous (MC) manifestations.

Note – *VL recurrence after first treatment. “—” refers to the absence of data.

Furthermore, the 4 observational studies docu-mented in the literature, gathered in Table 2, provide data from 66 patients in total. The patients in Tables 1 and 2 were of an average age of 42.52 (18–75) years; 78 of the patients (85.71%) were male.

**Table 2:** Data from observational studies (≥8 patients) of visceral leishmaniasis among kidney transplant recipients.

	Oliveira et al. (2008)^38^	Basset et al. (2005)^37^	Da Silva et al. (2013)^36^	De Silva et al. (2015)^35^
Patients	(n = 8)	(n = 8)	(n = 20)	(n = 30)
Location	Ceará (Brazil)	France	Ceará/Piauí (Brazil)	Ceará/Piauí (Brazil)
Male (%)	87.5	50	90	80
Average age	35.5 ± 11.2(22−57)	52.8 ± 8.1(38−67)	37 ± 10.7(18–60)	40 ± 10.5(22−60)
Previous blood transfusion (%)	–	–	50	43.3
Fever (%)	100	75	100	70
Splenomegaly (%)	100	12.5	100	93.3
Hepatomegaly (%)	100	12.5	–	70
Weight loss (%)	100	62.5	100	100
Skin Lesions (%)	–	–	80	83.3
Bone Marrow Microscopy+ (%)	87.5	75	95	63.3
rK39+ (%)	37.5	–	20	16.7
Cure (%)	100	87.5	85	80
VL remission with dialysis (%)	–	–	10	–
Relapse (%)	37.5	12.5	10	26.7
Death (%)	0	12.5 (refused treatment)	15	16.7
Treatment-associated nephrotoxicity	37.5% (before treatment, mean SCr was 2.58)	–	95% with SCr >30% elevated	On the 2^nd^ day of VL remission, the mean SCr value reached 2.21

Abbreviations – VL: visceral leishmaniasis; SCr: serum creatinine (mg/dL) at diagnosis.

### Asymptomatic Infection by Leishmania spp. in KT Candidates

In total, 1,348 KT candidates were included in the studies shown in Table 3, all from endemic areas for VL. Studies that compared diagnostic methods had conflicting results^5, 29, 30^. Furthermore, among the 1,348 patients, 188 (13.9%) tested positive in at least one of the diagnostic tests. Three studies explored the exposure of patients to blood transfusion^29, 30, 32^. In these studies, 34.8% of the 89 infected patients had undergone previous blood transfusions, and 14.1% of the 240 patients who received transfusions had positive serology for *Leishmania spp*.

**Table 3:** Data from observational studies on asymptomatic*Leishmania spp.*infection in KT candidates

	Country	Nº of patients	Average age (years)	Male (%)	Received transfusion	Test results (positivity)	Positive test result and past transfusion
Comai et al. (2021)^5^	Italy	119	70±13.55 (20-94)	61	—	19 (16.0%): 17 WB+ and 3 PCR+ (one patient WB and PCR)	—
Menike et al. (2022)^29^	Sri Lanka	124	44.48±11,36 (18-72)	81.4	115	4 (2.4%): 2 DAT+ and 2 rK39+	4 (100%)
França et al. (2020)^30^	Brazil	50	32±10 (20-72)	60	30	16 (32.0%): all IFAT+	10 (62.5%)
Deni et al. (2024)^31^	Italy	120	—	64.1	—	50 (41.7%): 9 WB+, 32 WBA+, and 4 PCR+	—
Souza et al. (2009)^32^	Brazil	310	—	52.9	81	69 (22.3%): all IFAT+	17 (24.6%)
Elmahallawy et al. (2015)^33^	Spain	625	49 (11–81)	64.0	—	30 (4.8%): all IFAT+	—

Abbreviations - KT: Kidney Transplant. VL: Visceral Leishmaniasis. L-AMB: Liposomal Amphotericin B. PAHO: Pan American Health Organization.

### Clinical and Laboratory Data

As seen in Table 1, the average time of symptom onset was 54.7 months post-transplant. Most cases had symptoms within less than 5 years post-transplant (60.6%). Only 4 cases reported the interval between symptom onset and VL diagnosis, with an average of 16 days (variation of 7–28 days)^9, 25, 26, 27^.

**Figure 2:**
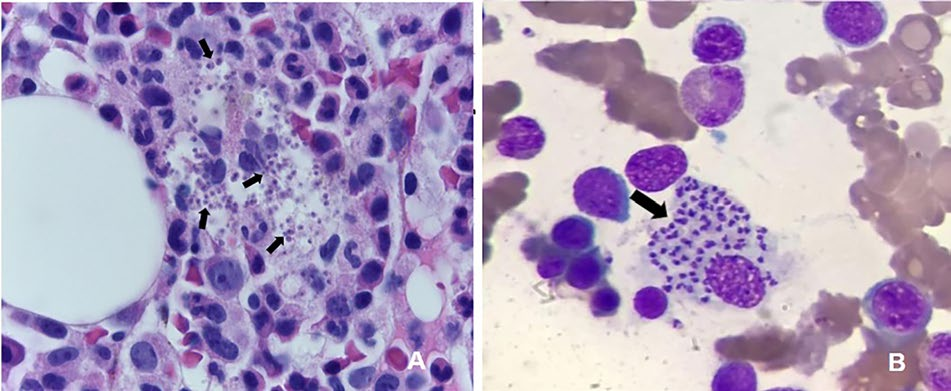
Bone marrow biopsy (A) and aspirate (B) (hematoxylin-eosin (H&E), 1000x) showing interstitial macrophages containing numerous rounded microorganisms (amastigotes) in the cytoplasm (arrows), compatible with *Leishmania spp*. (Fazzio CSJ and Farias LABG with permission).

In the 25 case reports, the main diagnostic tests utilized were: bone marrow microscopy (73.9% positivity - illustrated in Figure 2); PCR (100%); and serology methods (77.8%). Only one study employed rK39 testing^29^. These data are not presented in Table 1.

Symptoms and laboratory tests were analyzed in each patient of Table 1: thrombocytopenia (84%), leukopenia (84%), bicytopenia (84%), anemia (80%), pancytopenia (80%), fever (65.7%), splenomegaly (64%), fatigue (44%), weight loss (40%)^10, 12, 13, 14, 18, 22, 25, 28^ hepatomegaly (24%), anorexia (20%), diarrhea (16%)^10, 19, 22, 38^, and lymphadenopathy (16%)^12, 16, 17, 24, 38^ were common findings. The prevalence of the main symptoms mentioned in the large cohorts is detailed in Table 2. Furthermore, in some cases, there were associations between VL and cutaneous (12%)^11, 17, 20^ and/or mucocutaneous (16%)^11, 15, 17, 24^ forms.

In terms of mortality associated with VL, taking into consideration a maximum of 2 years between first diagnosis and death, a rate of 20% was found for the cases in Table 1, and varied from 0–16.7% for the cases in Table 2. In Table 1, for patients who developed only VL, the mortality rate was 15%, while for those who presented either the cutaneous or mucocutaneous form, a mortality rate of 33.3% was found.

### Kidney Biopsy Findings

Only 5 (20.0%) of the 25 patients in Table 1 underwent a kidney biopsy, and two of them were due to kidney injuries not associated with VL^17, 19^. Among the three patients with renal damage attributable to VL, the following findings were observed: diffuse interstitial fibrosis with tubular atrophy, moderate chronic interstitial inflammation, glomerulosclerosis, chronic vascular damage, and identification of *Leishmania spp.* kinetoplastic DNA (kDNA), without amastigotes^9^; glomerulosclerosis, along with moderate diffuse inflammation, in addition to amastigotes inside macrophages^12^; and chronic nephropathy, interstitial fibrosis and tubular atrophy, along with segmental and focal glomerulosclerosis (SFGS)^23^.

### Management

As shown in Table 1, the treatment of choice for VL in KT recipients was L-AmB, due to its lower risk of nephrotoxicity and effectiveness^18^, with doses ranging from 3 to 5 mg/kg and a duration ranging from 5 to 10 days. Only one patient received secondary prophylaxis after the initial diagnosis with monthly L-AmB dose of 3 mg/kg^12^. In cases of relapse, a new regimen with L-AmB was initiated. In case of intolerance or therapeutic failure, patients were switched to alternative medications (glucantime, pentamidine or miltefosine)^12, 17, 19, 23^. In the remaining cases, treatment started with second-choice drugs, due to the unavailability of L-AmB (more expensive)^26, 38^. L-AmB usually provides a transient increase in SCr^36^. Seventy-two percent of patients in Table 1 (who reported renal function) had an increase in SCr during or after treatment with L-AmB. However, some patients already had changes in kidney function with symptoms since admission, and in some cases there was no report on kidney function after the adopted therapy.

Six (24%) out of 25 patients from Table 1 reported nephrotoxicity^9, 12, 19, 21, 23, 25^. Four of them were treated primarily with L-AmB. In one case, L-AmB was delayed^25^ and in another, glucantime was used as prophylaxis^23^. Two patients presented pancreatitis attributed to pentavalent antimony^13, 23^.

### Relapses

Relapse was defined as a recurrence of signs and symptoms from 1–24 months after completing successful therapy^39^. Of the 25 cases in Table 1, recurrences were observed in 3 cases (12%). Of the cases with recurrences, 1 (33.3%) progressed to death^21^. Notably, one case had five relapses^23^, eventually leading to death^18^. Only one of the patients with relapses started secondary prophylaxis, with L-Amb at a dose of 4 mg/kg for 4 weeks after the first recurrence^18^. In Table 2, a total of 66 patients are reported, and the relapse rate ranged from 10–37.5%.

## Discussion

In this review, KT recipients with VL were from or had traveled to countries classified as leishmaniasis-endemic by the World Health Organization (WHO)^40^. However, with climate change, the concept of “endemic country” may vary^41^. In transplant patients, VL can develop through three main mechanisms: (1) new infection in an immunocompromised recipient, especially after travelling to endemic regions; (2) reactivation of an asymptomatic infection triggered by immunosuppressive drugs; and (3) iatrogenic transmission via the transplanted organ^42, 43^ or blood products^4^.

Table 3 shows the prevalence of asymptomatic carriers among KT candidates^8^. In the present study, the frequency of asymptomatic infection in KT candidates was not significantly higher than the general population (13.9% in Table 3 vs. 11.2% in meta-analysis^44^). The current guidelines do not recommend serological screening for asymptomatic infection in organ donors, transplant recipients, or candidates, including those in endemic countries^6, 45, 46, 47^. However, this is a controversial topic, and the lack of agreement on methods, the inability to distinguish previous exposure from active infection, and the potential cross-reaction with other protozoa all limit the use of serological screening^6^. Nevertheless, if an available donor is known to be seropositive, it is advisable to perform clinical and laboratory monitoring of the recipient in the post-transplant period rather than to reject the organ for transplant (Table 4)^45^. Table 3 also presents test results (serological and molecular) used for screening, and there is a notable discrepancy in methods and results^5, 31^. For this reason, this review was not designed to compare screening methods. Sensitivity and specificity vary according to the methods chosen, the antigens used, and the geographical area^6, 51^. Nevertheless, different methods have been proposed^31, 52^, and results usually present high positivity^4^, although prospective studies with a greater number of patients from endemic and non-endemic areas are still needed.

**Table 4:** Visceral leishmaniasis management in the context of kidney transplantation

Situation	Formal Recommendation	Explanation/Discussion
Screening for *Leishmania spp.* in blood banks	Not included in systematic screening in blood banks^2, 48^.	There are no gold-standard methods established for screening of asymptomatic infection^49^. Leukoreduction has been proposed to reduce transfusion-transmitted leishmaniasis^2^.
Screening for VL in donor and recipient	Not included in routine systematic screening. A known asymptomatic infection in the donor should not reject the organ for transplant^45^	There are no gold-standard methods established for screening of asymptomatic infection^7, 49^. Proven transmission through organ grafts is exceptional^42, 43^, therefore, routine screening of donors is not recommended^49^. However, a known positive serology of the organ donor or recipient may indicate a closer follow-up and early treatment of the recipient, if disease is suspected^45^.
Primary prophylaxis for VL among KT recipients	Neither primary prophylaxis nor preventive treatment is recommended in asymptomatic patients^7, 45^.	—
Secondary prophylaxis for VL among KT recipients	Secondary prophylaxis with L-AmB can prevent relapses in recurrent cases^7, 45^. It should be evaluated on a case-by-case basis, and frequent clinical follow-up is recommended^50^.	Posology: L-AmB, 3 mg/kg/dose every 2–3 weeks^45, 50^.
Preferred treatment for VL in KT recipients	L-AmB is the drug of choice, along with immunosuppression reduction during treatment^3, 7, 45^.	Food and Drug Administration: 4 mg/kg/day IV in days 1–5, 10, 17, 24, 31 e 38 (total dose of 40 mg/kg)^45^. PAHO: 3 mg/kg/day IV up to 20–40 mg/kg total dose^50^.
Management of VL treatment-associated nephropathy	Premedication; saline loading; dose testing; slow infusions (2–6 h); electrolyte supplementation, increased intervals between doses, and/or drug holidays, if indicated^45^. Avoid/minimize use of other nephrotoxic agents ^45^.	PAHO guidelines emphasize strict renal function monitoring during treatment, especially in immunocompromised patients^50^.

Notes - VL infection was considered based on a positive result in any of the tests. RK39+: number of patients who tested positive for RK39-ELISA. DAT+: number of patients who tested positive for direct agglutination test. WB+: number of patients who tested positive for specific igg western-blot method ANTI-*LEISHMANIA*. PCR+: number of patients who tested positive for dna polymerase chain reaction of the *LEISHMANIA SPP.* KINETOPLAST*.* IFAT+: Number Of Patients Who Tested Positive For ANTI*-LEISHMANIA SPP.* Antibody Immunofluorescence Test. WBA+: number of patients who tested positive for WHOLE BLOOD ASSAY.

Cytokine release assays (such as the interferon gamma release assay - IGRA) have been increasingly studied to detect asymptomatic infection and clinical cure after treatment of patients receiving immunosuppressive agents^53^. To the best of our knowledge, only one such study shows promising accuracy for this screening method, and its clinical applicability remains uncertain^54^.

A prospective cohort suggests that the screening of donors and recipients could be performed in cases at high risk of transmission or reactivation, and PCR could thus be a tool with greater specificity^46^. Nonetheless, this study is small, PCR positivity may be transient, and a positive result in the recipient does not necessarily predict disease development^46^. One case report of a lung transplant recipient shows that VL could have been diagnosed months before the development of symptoms by quantitative PCR, suggesting its role in early diagnosis^55^.

In three cohorts (see Table 3), 34.8% of infected KT candidates had a history of transfusion, and 14.1% of the blood transfused patients tested positive for *Leishmania spp* ^29, 30, 32^. Although rare, transfusion-transmitted leishmaniasis has been previously described in the literature^56, 57, 58^, and blood transfusions may be a source of infection for KT candidates and recipients^13^. The occurrence of previous transfusions in these studies remains to be detailed, given their well-established role in the pathogenesis of leishmaniasis^4, 57^. Current guidelines, including the WHO, do not universally recommend screening for VL in blood banks, even in endemic regions (Table 4)^48^.

Tables 1 and 2 focus on clinical data. Fever, weight loss, splenomegaly, and pancytopenia are considered classic symptoms of VL, but may not always be present. Atypical manifestations may delay diagnosis^46^. In a recent large systematic review of *Leishmania* infection in KT recipients, classic manifestations were present in more than 90% of patients^1^. Nevertheless, the clinician needs to be aware of atypical presentations. Furthermore, serology should always be used at least as a first-line method of diagnosis in transplant recipients whenever VL is suspected^4^. It is possible that the inhibition of cellular immunity may affect clinical presentation and outcomes, as suggested in patients with co-infection with VL and acquired immunodeficiency virus (HIV)^59, 60^, but experimental studies to support this hypothesis are seriously lacking in the SOT population. Despite that, it is formally suggested to reduce immunosuppression during VL treatment^1, 45^, as with any opportunistic infection. A higher mortality is observed in this group of patients. The rate can be increased when VL is associated with a cutaneous or mucosal presentation (33.3% vs. 15% of mortality in isolated VL, in Table 1). The reason for this is not fully understood, but it is reasonable to consider a more severe and disseminated disease.

**Figure 3:**
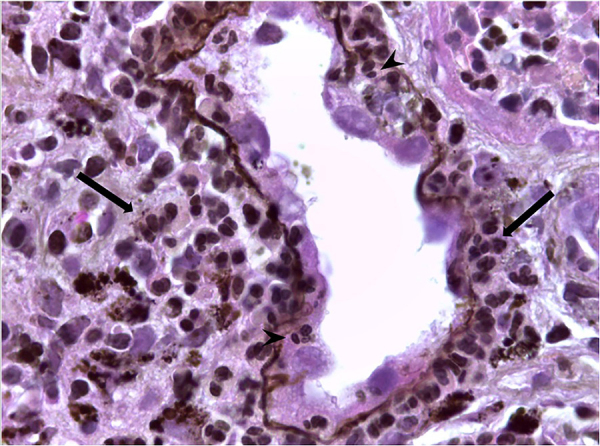
Renal biopsy showing visceral leishmaniasis-associated nephropathy (Methenamine Silver, 400x). Tubule and interstitium with inflammatory infiltrate, mostly composed of lymphocytes and macrophage (larger arrows); acute tubular epithelial degenerative changes and tubulitis (smaller arrows). (Baptista MASF with permission).

The impact of VL in renal function is presented in Tables 1 and 2, with increased levels of SCr at diagnosis. A retrospective study found an increase of more than 30% in SCr in 95% of KT recipients with VL^36^. Irreversible renal dysfunction was a rare event, occurring in only one patient, and was also associated with antimony nephrotoxicity^23^. Kidney damage has a complex physiopathology. Francesco Daher et al.^61^ describe the formation of systemic and *in situ* immune complexes in leishmaniasis nephropathy, emphasizing their interaction with glomerular antigens and the involvement of inflammatory cells. Figure 3 shows some patterns of leishmaniasis nephropathy. The biopsy aims to determine the cause of kidney injury, but it is challenging to distinguish between the effects of parasitic infection, drug-induced nephrotoxicity, or graft rejection. Glomerular lesions (especially FSGS) and tubulointerstitial nephritis are common patterns in VL nephropathy^62^. Additionally, in this review, interstitial involvement was also common and associated with mononuclear infiltrate and tubular atrophy^61^. Although uncommon^63^, parasites were seen in a kidney biopsy of one of the cases^12^.

Overall, immunosuppression should be reduced during the anti-VL treatment, but this depends on a case-by-case assessment^7, 45^. Indications for primary and secondary prophylaxis are not yet defined, but possible approaches are presented in Table 4 ^45, 50^. There is a scarcity of randomized controlled trials and meta-analyses of VL treatment, especially in immunosuppressed populations without HIV, and guidelines are based on extrapolations of reports and small case series^50^. Current treatment recom-mendations are mostly based on expert opinion, but the preferred treatment option is L-AmB (Table 4)^7, 45, 50^. However, it is occasionally necessary to resort to alternative medications due to either intolerance or lack of availability of L-AmB, and patients should be carefully monitorized^7, 45, 50^. Nephrotoxicity is a major concern in these patients due to pre-existing kidney function impairment. L-AmB is preferred over amphotericin deoxycholate for its lower toxicity^7, 45^. The management and monitoring of L-AmB nephrotoxicity are shown in Table 4 ^7, 19^. Other drug options may be considered^7^.

Moreover, 12% of patients experienced relapses, with a mortality rate of 33.3% (Table 1). Relapse rates in larger series were similar, ranging from 10% to 37.5% (Table 2)^4^. Monitoring immunosuppressed patients with VL for relapses is recommended for at least one year following diagnosis^4, 45^. This follow-up should be guided by clinical and laboratory assessments, depending on availability^45, 50^.

This review has inherent limitations due to its retrospective nature and reliance on the quality of clinical records for data accuracy. The studies included were conducted over an extended period of time, potentially leading to variability in findings. Additionally, some cases may have been duplicated in individual case reports and larger series. The aim of this review was to focus in VL within the KT scenario, but there is a scarcity of studies on asymptomatic candidates and the impact of previous infection. The lack of uniformity among these studies limits direct comparisons. The role of immunosuppression on the serological diagnosis and outcomes of VL in SOT recipients is also challenging, particularly due to the lack of immunological studies. Finally, given that VL in KT recipients is a rare occurrence, even in endemic regions, the statistical power of the conclusions of this review is significantly limited. Nonetheless, the study highlights important considerations regarding screening and the risk of disease development in this specific group of patients. Although VL is rare among transplant recipients and often neglected even in endemic areas, early identification and appropriate treatment are crucial for improving survival outcomes.

## Conclusion

This integrative review assessed the clinical profile of VL in KT recipients and candidates, emphasizing the limited data available for this group. Most VL cases displayed typical presentations, although atypical forms were challenging to diagnose. Concomitant mucocutaneous involvement was associated with higher mortality rates. Serological tests are not routinely performed for the screening of asymptomatic infection, and KT candidates did not show a higher prevalence of latent infection compared to the general population. While renal function may decline due to the disease or its treatment, graft loss remains uncommon. Blood transfusions could increase the risk of infection, but routine screening in blood banks for donors or recipients is not currently recommended. Further studies are needed in order to better understand the role of immunosuppression and secondary prophylaxis in VL management.

## Data Availability

The data that support the findings of this study are available upon request and in the manuscript tables.
