# Supplementary material for: Visceral leishmaniasis in kidney transplant recipients and candidates: an integrative review of the last 20 years
Source: J Bras Nefrol. 2025 May 19;47(2):e20240138. doi: 10.1590/2175-8239-JBN-2024-0138en (PMC12088644; doi:10.1590/2175-8239-JBN-2024-0138en)
Supplement: Translation

# Translation: Leishmaniose visceral em receptores e candidatos a transplante renal: uma revisão integrativa dos últimos 20 anos

**DOI:** 10.1590/2175-8239-JBN-2024-0138pt

**Authors:** Osvaldo Mariano Viana, Ednaldo Pereira Lima, Beatriz Fontenele Felix, Gustavo Ferreira da Silva, Pedro Yago Lima de Mesquita, Francisco Mikael Alves Mota, Evelyne Santana Girão, Maria Alice Sperto Ferreira Baptista, Celia Sebastiana de Jesus Fazzio, Elizabeth De Francesco Daher, Wanessa Trindade Clemente

**Affiliations:** 1 Universidade Federal do Ceará, Faculdade de Medicina, Departamento de Medicina Clínica, Fortaleza, CE, Brasil.; 2 Universidade Federal do Ceará, Hospital Universitário Walter Cantídio, Fortaleza, CE, Brasil.; 3 Hospital de Base de São José do Rio Preto, São José do Rio Preto, SP, Brasil.; 4 Universidade Federal de Minas Gerais, Faculdade de Medicina, Departamento de Propedêutica Complementar, Belo Horizonte, MG, Brasil.

**Keywords:** Transplante de Rim, Transplante Renal, Leishmaniose Visceral, Doenças Negligenciadas, Hospedeiro Imunocomprometido

## Abstract

**Introdução:**

A leishmaniose é uma possível preocupação para receptores de transplante de órgãos sólidos (TOS), especialmente aqueles provenientes de regiões endêmicas. Dentre os procedimentos de TOS, o transplante renal (TR) é o mais comum. Este estudo tem como objetivo sintetizar as evidências sobre leishmaniose visceral (LV) em candidatos e receptores de TR, com foco nos fatores de risco e desfechos associados.

**Metodos:**

Esta revisão integrativa analisou estudos dos últimos 20 anos, concentrando-se no perfil da doença, tratamento, prognóstico e risco de infecção assintomática.

**Resultados:**

Foram incluídos 32 artigos. Dos receptores de TR, 85,7% eram do sexo masculino, com idade média de 42,5 anos. O tempo médio desde o início dos sintomas foi de 54,7 meses. O comprometimento da função renal foi relatado em 64% dos pacientes, com uma taxa de mortalidade associada de 15%. A recidiva pós-tratamento ocorreu em 10-37,5% dos pacientes. Entre os candidatos ao TR, 13,9% apresentaram soropositividade para Leishmania spp.

**Conclusão:**

A LV é uma condição pouco frequente entre receptores de TR, o que limita a qualidade das evidências disponíveis. A detecção precoce e o tratamento imediato são cruciais para a melhoria dos desfechos. Embora o comprometimento da função renal seja comum, ele raramente leva à rejeição do enxerto. Nos estudos revisados, a coexistência de LV e formas cutâneas ou mucocutâneas esteve associada a maior mortalidade. As recidivas são comuns e exigem estratégias de manejo individualizadas. A hemotransfusão representa um potencial risco de infecção, embora a triagem de rotina em bancos de sangue ainda não seja recomendada.

## Introdução

A leishmaniose visceral (LV) é uma doença parasitária tropical negligenciada, causada por um grupo de protozoários intracelulares pertencentes ao complexo *Leishmania donovani*, também conhecido como “calazar”. Esses agentes patogênicos, transmitidos pela picada da fêmea do mosquito *Phlebotomus* ou *Lutzomyia*, apresentam tropismo por células reticuloendoteliais, principalmente as do baço, fígado e medula óssea^1^.

Em receptores de transplante, o uso de agentes imunossupressores é essencial para prevenir a rejei-ção do enxerto, que está associada à manifestação sintomática da infecção por mecanismos ainda não totalmente elucidados e que podem reativar infecções assintomáticas^2, 3, 4^. Os receptores de transplante renal (TR) podem ser infectados por meio de transfusão sanguínea, transmissão pelo aloenxerto ou exposição posterior a flebotomíneos infectados^4^. Informações sobre a LV assintomática em candidatos a transplante são escassas, e o reconhecimento da doença no receptor do transplante pode, portanto, sofrer atrasos^5^.

O diagnóstico envolve a identificação de parasitas principalmente por microscopia (da medula óssea e, menos frequentemente, do baço). Também podem ser utilizados o isolamento da cultura, o teste imunocromatográfico rápido com rK39 (detecção de antígeno) e a reação em cadeia da polimerase (PCR) a partir do sangue periférico ou da medula óssea^6^. A sorologia, por meio do teste de imunofluorescência indireta (IFI), do ensaio de imunoabsorção enzimática (ELISA) e do teste de aglutinação direta (TAD), é uma opção, embora cada teste possa apresentar diferentes níveis de sensibilidade e especificidade^6^. O tratamento preferido para esses pacientes requer uma abordagem sistêmica, sendo geralmente recomendável uma redução da dose de imunossupressão. O medicamento de escolha é a anfotericina B lipossomal (L-AmB) e, alternativamente, os antimoniais pentavalentes, que podem causar danos renais. Outras opções menos comuns incluem a miltefosina, pentamidina e paromomicina^7^.

A LV é uma infecção incomum no período pós-transplante, mesmo em regiões endêmicas. Entre os receptores de transplante de órgãos sólidos (TOS), a LV é relatada com mais frequência no TR, sendo responsável por 77% dos casos^1^. Esse achado provavelmente reflete a maior prevalência desse tipo de transplante entre os TOS^4, 8^. Normalmente, a infecção por LV causa febre, pancitopenia, hepatoesplenomegalia e perda de peso. No entanto, essas são as manifestações clássicas, mas não universais, da LV. Em alguns casos, a doença também pode levar à nefropatia, causada por infiltração inflamatória e esclerose glomerular, o que pode levar à disfunção do enxerto em receptores de TR^1, 5, 9^.

Este estudo teve como objetivo elucidar o conhecimento científico atual sobre a infecção por leishmaniose e os desfechos entre candidatos e receptores de TR, enfatizando os efeitos adversos relacionados ao tratamento e à saúde renal nessa população.

## Métodos

Foi realizada uma revisão integrativa para discutir o perfil clínico e o diagnóstico da infecção por LV em candidatos e receptores de transplante renal, além de delinear os tratamentos e fatores prognósticos.

A pesquisa foi realizada usando os descritores “(‘Leishmaniose Visceral’) E (‘Transplante de Rim’ OU ‘Transplante Renal’)” em três bancos de dados (PubMed, *Google Scholar* e MEDLINE) em março de 2024. A busca abrangeu o período de 2004 a 2024, selecionando apenas artigos em inglês. Foram incluídos relatos e séries de casos, coortes retrospectivas e revisões sistemáticas. As duplicatas foram removidas manualmente e os resumos de conferências foram excluídos.

Os critérios de inclusão foram estudos que descrevessem pacientes com LV ou infecção assintomática por *Leishmania* spp. diagnosticadas em receptores e candidatos a TR, publicados em periódicos indexados. Os critérios de exclusão foram estudos focados em formas não viscerais de leishmaniose (cutânea e mucocutânea), aqueles envolvendo pacientes não receptores de TR ou outros tipos de transplante que não o TR (por exemplo, transplantes de fígado ou de células-tronco hematopoiéticas) e manuscritos que não especificaram o tipo de transplante.

O processo de seleção de artigos foi realizado por dois autores (OMVN e PYLM), e quaisquer incertezas quanto à inclusão ou exclusão foram discutidas no grupo de autores para consenso. A lista final de artigos selecionados foi documentada em uma planilha, que foi gerenciada pelos autores, para análise de dados subsequente.

## Resultados

### Estudos Incluídos

A Figura 1 mostra um fluxograma que detalha o processo de seleção dos estudos incluídos nesta revisão.

**Figura 1:** Fluxograma de seleção dos estudos. Fluxograma do *Preferred Reporting Items for Systematic Reviews and Meta-Analyses* (PRISMA) adaptado para revisões integrativas.

Os autores categorizaram os manuscritos em subgrupos da seguinte forma: relatos ou séries de casos sobre receptores de TR^9, 10, 11, 12, 13, 14, 15, 16, 17, 18, 19, 20, 21, 22, 23, 24, 25, 26, 27, 28^; estudos observacionais sobre infecção assintomática em candidatos a TR^5, 29, 30, 31, 32, 33^; estudos observacionais sobre pacientes de TR que desenvolveram LV^34, 35, 36, 37, 38^ e uma revisão sistemática da LV em receptores de TR^1^.

A epidemiologia da LV em receptores de TR, a prevalência de infecção assintomática em candidatos a TR, os dados clínicos e laboratoriais, os achados de biópsia, o manejo terapêutico e as recidivas foram observados em cada um dos estudos incluídos.

### Epidemiologia

A seleção de artigos resultou em 20 publicações de relatos de casos e séries de casos contendo dados de 25 pacientes (Tabela 1). A maioria dos relatos é originária de países endêmicos, como os da Bacia do Mediterrâneo, Brasil, Índia, Arábia Saudita e Irã^13^. Três pacientes relataram viagens: um para a Espanha e Tunísia^12^, um para a França e Marrocos^19^ e um para o Brasil e Tailândia^25^.

**Tabela 1:** Dados de relatos de caso e pequenas séries de caso (< 8 pacientes) de leishmaniose visceral em transplantados renais.

	Sintomas	Tempo após TR	CrS	Apresentação LC/LM	País	Óbito	Tratamento
Busutti et al.^9^, 2023	Suor noturno e febre	18	3,7	Nenhuma	Itália	Não	L-AmB
Rana et al.^10^, 2022	Dor abdominal e perda de peso	48	2,5	Nenhuma	Índia	Não	L-AmB
Marques et al.^11^, 2020	Febre e úlceras bucais	108	–	Ambas	Portugal	Sim	L-AmB
Dettwiler et al.^12^, 2010	Febre, anorexia, perda de peso, astenia e hepatoesplenomegalia	69	2,71	Nenhuma	Viagem para área endêmica	Sim	L-AmB
Bouchekoua et al.^13^, 2014	Febre, anorexia, astenia e perda de peso	17	4,18	Nenhuma	Tunísia	Não	Glucantime
Kardeh et al.^14^, 2023	Febre, calafrios e mal-estar	23	1,61	Nenhuma	Irã	Não	L-AmB
Madhyastha et al.^15^, 2016	Febre e úlceras bucais	62	4,3	LM	Índia	Não	L-AmB
Sánchez et al.^16^, 2018 **(Caso 1)**	Febre e adenopatias	24	–	Nenhuma	Espanha	Não	L-AmB
Sánchez et al.^16^, 2018 **(Caso 2)**	Febre	192	–	Nenhuma	Espanha	Não	L-AmB
Simon et al.^17^, 2011 **(Caso 1)**	Úlceras bucais	120	–	LM	Itália	Sim	L-AmB
Simon et al.^17^, 2011 **(Caso 2)**	Febre e deterioração geral	204	–	LC	Itália	Não	L-AmB*
Zumrutdal et al.^18^, 2010	Febre, hepatoesplenomegalia e perda de peso	60	2,2	Nenhuma	Turquia	Não	L-AmB/ Alopurinol*
Duvignaud et al.^19^, 2015	Febre, astenia e diarreia	72	1,54	Nenhuma	Viagem para área endêmica	Não	L-AmB/Pentamidina
Yücel et al.^20^, 2013	Febre e pápulas cutâneas	84	–	LC	Turquia	Sim	L-AmB
Pedroso et al.^21^, 2014	Febre, calafrios e indisposição	192	–	Nenhuma	Itália	Sim	L-AmB*
Oliveira et al.^22^, 2008 **(Caso 1)**	Febre, astenia, anorexia e diarreia	5	1,8	Nenhuma	Brasil	Não	L-AmB
Oliveira et al.^22^, 2008 **(Caso 2)**	Febre, calafrios, anorexia e perda de peso	36	2,5	Nenhuma	Brasil	Não	L-AmB
Oliveira et al.^22^, 2008 **(Caso 3)**	Febre, anorexia, astenia, linfadenopatia e hepatoesplenomegalia	36	1,2	Nenhuma	Brasil	Não	L-AmB
Oliveira et al.^22^, 2008 **(Caso 4)**	Diarreia, perda de peso, dor abdominal, astenia, anorexia e febre	8	2,7	Nenhuma	Brasil	Não	L-AmB
Rancan et al.^23^, 2022	Diarreia, febre e hepatoesplenomegalia	48	–	Nenhuma	Brasil	Sim	L-AmB
Jha et al.^24^, 2012	Febre, linfadenopatia cervical e esplenomegalia	84	1,1	LM	Nepal	Não	L-AmB
Pêgo et al.^25^, 2013	Febre, indisposição, fraqueza, suor noturno e caquexia	17	1,48	Nenhuma	Viagem para área endêmica	Não	L-AmB
Prasad et al.^26^, 2011	Febre, astenia e mialgia	84	5,2	Nenhuma	Índia	Não	L-AmB
Gembillo et al.^27^, 2021	Anemia, febre e retenção urinária	36	4	Nenhuma	Itália	Não	L-AmB
Keitel et al.^28^, 2018	Febre, mialgia, perda de peso, fraqueza e esplenomegalia	33	–	Nenhuma	Brasil	Não	L-AmB

Abreviações – TR: transplante renal; CrS: creatinina sérica (mg/dL) no diagnóstico; LC/LM: presença de manifestações cutâneas (LC) e mucocutâneas (LM).

Nota – *Recidiva de LV após o primeiro tratamento. “—” refere-se à ausência de dados.

Além disso, os quatro estudos observacionais documentados na literatura, reunidos na Tabela 2, fornecem dados de um total de 66 pacientes. Os pacientes das Tabelas 1 e 2 apresentavam uma idade média de 42,52 (18-75) anos; 78 deles (85,71%) eram do sexo masculino.

**Tabela 2:** Dados de estudos observacionais (≥8 pacientes) de leishmaniose visceral em receptores de transplante de rim.

	Oliveira et al. (2008)^38^	Basset et al. (2005)^37^	Da Silva et al. (2013)^36^	De Silva et al. (2015)^35^
Pacientes	(n = 8)	(n = 8)	(n = 20)	(n = 30)
Local	Ceará (Brasil)	França	Ceará/Piauí (Brasil)	Ceará/Piauí (Brasil)
Sexo masculino (%)	87,5	50	90	80
Idade média	35,5 ± 11,2(22−57)	52,8 ± 8,1(38−67)	37 ± 10,7(18–60)	40 ± 10,5(22−60)
Transfusão sanguínea prévia (%)	–	–	50	43,3
Febre (%)	100	75	100	70
Esplenomegalia (%)	100	12,5	100	93,3
Hepatomegalia (%)	100	12,5	–	70
Perda de peso (%)	100	62,5	100	100
Lesões Cutâneas (%)	–	–	80	83,3
Microscopia de Medula Óssea+ (%)	87,5	75	95	63,3
rK39+ (%)	37,5	–	20	16,7
Cura (%)	100	87,5	85	80
Remissão da LV com diálise (%)	–	–	10	–
Recidiva (%)	37,5	12,5	10	26,7
Óbito (%)	0	12,5 (recusou tratamento)	15	16,7
Nefrotoxicidade associada ao tratamento	37,5% (antes do tratamento, CrS média foi de 2,58)	–	95% com CrS >30% elevada	No 2º dia de remissão da LV, o valor médio de CrS atingiu 2,21

Abreviações – LV: leishmaniose visceral; CrS: creatinina sérica (mg/dL) no diagnóstico.

### Infecção Assintomática por *Leishmania spp*. em Candidatos a TR

No total, 1.348 candidatos a TR foram incluídos nos estudos exibidos na Tabela 3, todos provenientes de áreas endêmicas para LV. Estudos que compararam os métodos diagnósticos apresentaram resultados conflitantes^5, 29, 30^. Além disso, entre os 1.348 pacientes, 188 (13,9%) testaram positivo em pelo menos um dos testes diagnósticos. Três estudos investigaram a exposição dos pacientes a transfusões sanguíneas^29, 30, 32^. Nesses estudos, 34,8% dos 89 pacientes infectados haviam recebido transfusões de sangue anteriores, e 14,1% dos 240 pacientes que receberam transfusões apresentaram sorologia positiva para *Leishmania spp*.

**Tabela 3:** Dados de estudos observacionais sobre infecção assintomática por Leishmania spp. em candidatos ao transplante renal

	País	Nº de pacientes	Idade média (anos)	Sexo masculino (%)	Recebeu transfusão	Resultados dos testes (positividade)	Resultado positivo do teste e transfusão anterior	
Comai et al. (2021)^5^	Itália	119	70±13,55 (20-94)	61	—	19 (16,0%): 17 WB+ e 3 PCR+ (um paciente WB e PCR)	—
Menike et al. (2022)^29^	Sri Lanka	124	44,48±11,36 (18-72)	81,4	115	4 (2,4%): 2 TAD+ e 2 rK39+	4 (100%)
França et al. (2020)^30^	Brasil	50	32±10 (20-72)	60	30	16 (32,0%): todos IFAT+	10 (62,5%)
Deni et al. (2024)^31^	Itália	120	—	64,1	—	50 (41,7%): 9 WB+; 32 WBA+ e 4 PCR+	—
Souza et al. (2009)^32^	Brasil	310	—	52,9	81	69 (22,3%): todos IFAT+	17 (24,6%)
Elmahallawy et al. (2015)^33^	Espanha	625	49 (11–81)	64,0	—	30 (4,8%): todos IFAT+	—

Abreviações - TR: Transplante Renal. LV: Leishmaniose Visceral. L-AMB: Anfotericina B Lipossomal. OPAS: Organização Pan-Americana da Saúde.

### Dados Clínicos e Laboratoriais

Conforme observado na Tabela 1, o tempo médio para o início dos sintomas foi de 54,7 meses após o transplante. A maioria dos casos manifestou sintomas em menos de 5 anos pós-transplante (60,6%). Somente quatro casos relataram o intervalo entre o início dos sintomas e o diagnóstico de LV, com uma média de 16 dias (variação de 7 a 28 dias)^9, 25, 26, 27^.

Nos 25 relatos de casos, os principais testes diagnósticos utilizados foram: microscopia de medula óssea (73,9% de positividade - ilustrado na Figura 2); PCR (100%); e métodos sorológicos (77,8%). Apenas um estudo empregou o teste rK39^29^. Esses dados não são apresentados na Tabela 1.

**Figura 2.** Biópsia de medula óssea (A) e aspirado (B) (hematoxilina-eosina (H&E), 1000x) mostrando macrófagos intersticiais contendo numerosos microrganismos arredondados (amastigotas) no citoplasma (setas), compatíveis com *Leishmania spp*. (Fazzio CSJ e Farias LABG com permissão).

Os sintomas e os exames laboratoriais foram analisados em cada paciente da Tabela 1: trombocitopenia (84%), leucopenia (84%), bicitopenia (84%), anemia (80%), pancitopenia (80%), febre (65,7%), esplenomegalia (64%), fadiga (44%), perda de peso (40%)^10, 12, 13, 14, 18, 22, 25, 28^, hepatomegalia (24%), anorexia (20%), diarreia (16%)^10, 19, 22, 38^ e linfadenopatia (16%)^12, 16, 17, 24, 28^ foram achados comuns. A prevalência dos principais sintomas mencionados nas grandes coortes está detalhada na Tabela 2. Além disso, em alguns casos, houve associações entre LV e formas cutâneas (12%)^11, 17, 20^ e/ou mucocutâneas (16%)^11, 15, 17, 24^.

Com relação à mortalidade associada à LV, considerando um intervalo máximo de 2 anos entre o primeiro diagnóstico e o óbito, foi encontrada uma taxa de 20% para os casos da Tabela 1, variando de 0 a 16,7% para os casos da Tabela 2. Na Tabela 1, entre os pacientes que desenvolveram somente LV, a taxa de mortalidade foi de 15%, enquanto para aqueles que apresentaram a forma cutânea ou mucocutânea, a taxa de mortalidade encontrada foi de 33,3%.

### Achados de Biópsia Renal

Apenas 5 (20,0%) dos 25 pacientes da Tabela 1 foram submetidos à biópsia renal, e dois desses casos foram devidos a lesões renais não associadas à LV^17, 19^. Entre os três pacientes com dano renal atribuível à LV, foram observados os seguintes achados: fibrose intersticial difusa com atrofia tubular, inflamação intersticial crônica moderada, glomeruloesclerose, dano vascular crônico e identificação de DNA do cinetoplasto (kDNA) de *Leishmania spp.*, sem presença de amastigotas^9^; glomeruloesclerose associada à inflamação difusa moderada, além de amastigotas dentro de macrófagos^12^; e nefropatia crônica, fibrose intersticial e atrofia tubular, juntamente com glomeruloesclerose segmentar e focal (GESF)^23^.

### Manejo

Como demonstrado na Tabela 1, o tratamento de escolha para LV em receptores de TR foi a L-AmB, devido à sua eficácia e ao menor risco de nefrotoxicidade^18^, com doses que variam de 3 a 5 mg/kg e duração de 5 a 10 dias. Apenas um paciente recebeu profilaxia secundária após o diagnóstico inicial com dose mensal de L-AmB de 3 mg/kg^12^. Em casos de recidiva, um novo regime com L-AmB foi iniciado. Caso houvesse intolerância ou falha terapêutica, os pacientes trocavam o tratamento por medicamentos alternativos (glucantime, penta-midina ou miltefosina)^12, 17, 19, 23^. Nos demais casos, o tratamento foi iniciado com medicamentos de segunda escolha, devido à indisponibilidade da L-AmB (custo mais elevado)^26, 38^. A L-AmB geralmente provoca um aumento transitório na CrS36. Entre os pacientes da Tabela 1 (que relataram a função renal), 72% apresentaram um aumento na CrS durante ou após o tratamento com L-AmB. No entanto, alguns pacientes já tinham alterações na função renal com sintomas desde a admissão e, em alguns casos, não houve relato sobre a função renal após a terapia adotada.

Seis (24%) dos 25 pacientes da Tabela 1 relataram nefrotoxicidade^9, 12, 19, 21, 23, 25^. Quatro deles foram tratados principalmente com L-AmB. Em um caso, o uso de L-AmB foi adiado^25^ e, em outro, o glucantime foi utilizado como profilaxia^23^. Dois pacientes apresentaram pancreatite atribuída ao antimônio pentavalente^13, 23^.

### Recidivas

A recidiva foi definida como a recorrência de sinais e sintomas de 1 a 24 meses após a conclusão bem-sucedida da terapia^39^. Dos 25 casos da Tabela 1, foram observadas recidivas em 3 casos (12%). Entre esses, 1 (33,3%) evoluiu para óbito^21^. Notavelmente, um caso apresentou cinco recidivas^23^, que acabaram resultando em óbito^18^. Apenas um dos pacientes com recidivas iniciou a profilaxia secundária, com L-Amb na dose de 4 mg/kg por 4 semanas após o primeiro episódio^18^. Na Tabela 2, relata-se um total de 66 pacientes, com uma taxa de recidiva que variou de 10 a 37,5%.

## Discussão

Nesta revisão, os receptores de TR com LV eram provenientes de países classificados como endêmicos para leishmaniose pela Organização Mundial da Saúde (OMS) ou haviam viajado para essas regiões^40^. No entanto, com as mudanças climáticas, o conceito de “país endêmico” pode variar^41^. Em pacientes transplantados, a LV pode se desenvolver por meio de três mecanismos principais: (1) nova infecção em um receptor imunocomprometido, especialmente após viagens a regiões endêmicas; (2) reativação de uma infecção assintomática desencadeada por medicamentos imunossupressores; e (3) transmissão iatrogênica por meio do órgão transplantado^42, 43^ ou de hemoderivados^4^.

A Tabela 3 apresenta a prevalência de portadores assintomáticos entre os candidatos ao TR^8^. No presente estudo, a frequência de infecção assintomática em candidatos ao TR não foi significativamente superior à da população em geral (13,9% na Tabela 3 *vs.* 11,2% na meta-análise^44^). As diretrizes atuais não recomendam a triagem sorológica para infecção assintomática em doadores de órgãos, receptores ou candidatos ao transplante, incluindo aqueles em países endêmicos^6, 45, 46, 47^. No entanto, esse é um tópico controverso, e a falta de consenso sobre os métodos, a incapacidade de distinguir exposição prévia de infecção ativa e a possível reação cruzada com outros protozoários limitam o uso da triagem sorológica^6^. Não obstante, caso um doador disponível seja soropositivo, recomenda-se o monitoramento clínico e laboratorial do receptor no período pós-transplante em vez da rejeição do órgão para transplante (Tabela 4)^45^. A Tabela 3 também apresenta os resultados dos testes (sorológicos e moleculares) utilizados para triagem, sendo notável a discrepância existente entre os métodos e os resultados^5, 31^. Por esse motivo, esta revisão não foi desenhada para comparar métodos de triagem. A sensibilidade e a especificidade variam de acordo com os métodos escolhidos, os antígenos utilizados e a região geográfica^6, 51^. No entanto, diferentes métodos têm sido propostos^31, 52^ e os resultados geralmente apresentam alta positividade^4^, embora ainda sejam necessários estudos prospectivos com um número maior de pacientes de áreas endêmicas e não endêmicas.

**Tabela 4:** Manejo da leishmaniose visceral no contexto do transplante renal

Situação	Recomendação Formal	Explicação/Discussão
Triagem para *Leishmania spp.* em bancos de sangue	Não incluído na triagem sistemática em bancos de sangue^2, 48^.	Não existem métodos padrão-ouro estabelecidos para a triagem de infecção assintomática^49^. A leucorredução foi proposta para reduzir a leishmaniose transmitida por transfusão^2^.
Triagem para LV em doadores e receptores	Não incluído na triagem sistemática de rotina. Uma infecção assintomática conhecida no doador não deve ser motivo de rejeição do órgão para transplante^45^	Não existem métodos padrão-ouro estabelecidos para a triagem da infecção assintomática^7, 49^. A transmissão comprovada por meio de enxertos de órgãos é excepcional^42, 43^, e, portanto, a triagem de rotina em doadores não é recomendada^49^. No entanto, uma sorologia positiva conhecida do doador ou receptor do órgão pode indicar um acompanhamento mais próximo e tratamento precoce do receptor, caso haja suspeita de doença^45^.
Profilaxia primária para LV entre receptores de TR	Nem a profilaxia primária nem o tratamento preventivo são recomendados em pacientes assintomáticos^7, 45^.	—
Profilaxia secundária para LV entre receptores de TR	A profilaxia secundária com L-AmB pode prevenir recidivas em casos recorrentes^7, 45^. Ela deve ser avaliada caso a caso e recomenda-se o acompanhamento clínico frequente^50^.	Posologia: L-AmB, 3 mg/kg/dose a cada 2-3 semanas^45, 50^.
Tratamento de preferência para LV em receptores de TR	A L-AmB é o medicamento de escolha, juntamente com a redução da imunossupressão durante o tratamento^3, 7, 45^.	*Food and Drug Administration* (FDA): 4 mg/kg/dia IV nos dias 1-5, 10, 17, 24, 31 e 38 (dose total de 40 mg/kg)^45^. OPAS (Organização Pan-Americana da Saúde): 3 mg/kg/dia IV até 20-40 mg/kg de dose total^50^.
Manejo da nefropatia associada ao tratamento da LV	Pré-medicação; carga de solução salina; teste de dosagem; infusões lentas (2 a 6 horas); suplementação de eletrólitos, aumento dos intervalos entre as doses e/ou pausas entre os medicamentos, se indicado^45^. Evitar/minimizar o uso de outros agentes nefrotóxicos^45^.	As diretrizes da OPAS enfatizam o monitoramento rigoroso da função renal durante o tratamento, especialmente em pacientes imunocomprometidos^50^.

Notas - A infecção por LV foi considerada com base em um resultado positivo em qualquer um dos testes. RK39+: número de pacientes com teste positivo para RK39-ELISA. TAD+: número de pacientes que testaram positivo para o teste de aglutinação direta. WB+: número de pacientes que testaram positivo no método western-blot específico para IGG ANTI-*LEISHMANIA*. PCR+: número de pacientes com teste positivo para reação em cadeia da polimerase do DNA da *LEISHMANIA SPP.* CINETOPLASTO. IFAT+: número de pacientes com teste positivo para ANTI*-LEISHMANIA SPP.* Teste de imunofluorescência para anticorpos. WBA+: número de pacientes que testaram positivo para o hemograma completo (Whole Blood Assay).

Os ensaios de liberação de citocinas (como o ensaio de liberação de interferon gama - IGRA) têm sido cada vez mais estudados para detectar infecção assintomática e para a cura clínica após o tratamento de pacientes sob o uso de agentes imunossupressores^53^. Até onde temos conhecimento, apenas um desses estudos demonstrou precisão promissora para esse método de triagem, e sua aplicabilidade clínica permanece incerta^54^.

Uma coorte prospectiva sugere que a triagem de doadores e receptores poderia ser realizada em casos com alto risco de transmissão ou reativação, e a PCR poderia, portanto, ser uma ferramenta com maior especificidade^46^. No entanto, esse estudo possui uma amostra pequena, a positividade da PCR pode ser transitória e um resultado positivo no receptor não necessariamente prevê o desenvolvimento da doença^46^. Um relato de caso de um receptor de transplante pulmonar mostra que a LV poderia ter sido diagnosticada meses antes do desenvolvimento dos sintomas por meio da PCR quantitativa, sugerindo seu papel no diagnóstico precoce^55^.

Em três coortes (ver Tabela 3), 34,8% dos candidatos ao TR infectados tinham histórico de transfusão e 14,1% dos pacientes transfundidos testaram positivo para *Leishmania spp* ^29, 30, 32^. Embora rara, a leishmaniose transmitida por transfusão foi descrita anteriormente na literatura^56, 57, 58^ e as transfusões de sangue podem ser uma fonte de infecção para os candidatos e receptores de TR^13^. A ocorrência de transfusões anteriores nesses estudos ainda precisa ser detalhada, dado seu papel bem estabelecido na patogênese da leishmaniose^4, 57^. As diretrizes atuais, incluindo as da OMS, não recomendam universalmente a triagem para LV em bancos de sangue, mesmo em regiões endêmicas (Tabela 4)^48^.

As Tabelas 1 e 2 concentram-se nos dados clínicos. Febre, perda de peso, esplenomegalia e pancitopenia são considerados sintomas clássicos de LV, mas nem sempre estão presentes. Manifestações atípicas podem atrasar o diagnóstico^46^. Em uma ampla e recente revisão sistemática sobre a infecção por *Leishmania* em receptores de TR, as manifestações clássicas estiveram presentes em mais de 90% dos pacientes^1^. No entanto, o clínico precisa estar ciente das apresentações atípicas. Além disso, a sorologia deve sempre ser utilizada, pelo menos como método diagnóstico de primeira linha em receptores de transplante, sempre que houver suspeita de LV^4^. É possível que a inibição da imunidade celular possa afetar a apresentação clínica e os desfechos, conforme sugerido em pacientes com coinfecção por LV e vírus da imunodeficiência adquirida (HIV)^59, 60^, mas estudos experimentais para apoiar essa hipótese são seriamente escassos na população de TOS. Apesar disso, sugere-se formalmente a redução da imunossupressão durante o tratamento da LV^1, 45^, como ocorre com qualquer infecção oportunista. Uma maior mortalidade é observada nesse grupo de pacientes. A taxa pode ser aumentada quando a LV estiver associada a uma apresentação cutânea ou mucosa (33,3% *vs.* 15% de mortalidade na LV isolada, conforme Tabela 1). A razão para isso não é totalmente compreendida, mas é razoável considerar uma doença mais grave e disseminada.

O impacto da LV na função renal é apresentado nas Tabelas 1 e 2, com níveis aumentados de CrS no momento do diagnóstico. Um estudo retrospectivo encontrou um aumento de mais de 30% na CrS em 95% dos receptores de TR com LV^36^. A disfunção renal irreversível foi um evento raro, ocorrendo em apenas um paciente, e também foi associada à nefrotoxicidade do antimônio^23^. O dano renal possui uma fisiopatologia complexa. Francesco Daher et al.^61^ descrevem a formação de complexos imunes sistêmicos e *in situ* na nefropatia por leishmaniose, enfatizando sua interação com antígenos glomerulares e o envolvimento de células inflamatórias. A Figura 3 mostra alguns padrões da nefropatia por leishmaniose. A biópsia tem como objetivo determinar a causa da injúria renal, mas é desafiador distinguir entre os efeitos da infecção parasitária, da nefrotoxicidade induzida por medicamentos ou da rejeição do enxerto. As lesões glomerulares (especialmente a GESF) e a nefrite tubulointersticial são padrões comuns na nefropatia por LV^62^. Além disso, nessa revisão, o envolvimento intersticial também foi comum e associado a infiltrado mononuclear e atrofia tubular^61^. Embora incomum^63^, parasitas foram observados em uma biópsia renal de um dos casos^12^.

**Figura 3:** Biópsia renal mostrando nefropatia associada à leishmaniose visceral (metenamina de prata, 400x). Túbulo e interstício com infiltrado inflamatório, composto principalmente de linfócitos e macrófagos (setas maiores); alterações degenerativas epiteliais tubulares agudas e tubulite (setas menores). (Baptista MASF com permissão).

De modo geral, a imunossupressão deve ser reduzida durante o tratamento anti-LV, mas isso depende de uma avaliação caso a caso^7, 45^. As indicações para profilaxia primária e secundária ainda não estão definidas, mas possíveis abordagens são apresentadas na Tabela 4 ^45, 50^. Existe uma escassez de ensaios clínicos randomizados e meta-análises sobre o tratamento da LV, especialmente em populações imunossuprimidas sem HIV, e as diretrizes são baseadas em extrapolações de relatos e pequenas séries de casos^50^. As recomendações atuais de tratamento baseiam-se majoritariamente na opinião de especialistas, mas a opção de tratamento preferida é a L-AmB (Tabela 4)^7, 45, 50^. Entretanto, ocasionalmente, é necessário recorrer a medicamentos alternativos devido à intolerância ou à indisponibilidade de L-AmB, devendo os pacientes ser cuidadosamente monitorados^7, 45, 50^. A nefrotoxicidade é uma preocupação importante nesses pacientes devido ao comprometimento preexistente da função renal. A L-AmB é preferível ao desoxicolato de anfotericina por sua menor toxicidade^7, 45^. O manejo e o monitoramento da nefrotoxicidade da L-AmB são mostrados na Tabela 4 ^7, 19^. Podem ser consideradas outras opções de medicamentos^7^.

Além disso, 12% dos pacientes apresentaram recidivas, com uma taxa de mortalidade de 33,3% (Tabela 1). Em séries maiores, as taxas de recidiva foram semelhantes, variando de 10% a 37,5% (Tabela 2)^4^. Recomenda-se o monitoramento de recidivas em pacientes imunossuprimidos com LV por pelo menos um ano após o diagnóstico^4, 45^. Esse acompanhamento deve ser orientado por avaliações clínicas e laboratoriais, dependendo da disponibilidade^45, 50^.

Esta revisão apresenta limitações inerentes devido à sua natureza retrospectiva e à dependência da qualidade dos registros clínicos para a precisão dos dados. Os estudos incluídos foram realizados durante um longo período, o que pode levar a uma variabilidade nos achados. Além disso, alguns casos podem ter sido duplicados em relatos de casos individuais e séries maiores. O objetivo desta revisão foi focar na LV no contexto do TR, mas existe uma escassez de estudos sobre candidatos assintomáticos e o impacto de infecções prévias. A falta de uniformidade entre esses estudos limita comparações diretas. O papel da imunossupressão no diagnóstico sorológico e nos desfechos da LV em receptores de TOS também representa um desafio, especialmente devido à ausência de estudos imunológicos. Por fim, considerando que a LV em receptores de TR é uma ocorrência rara, mesmo em regiões endêmicas, o poder estatístico das conclusões desta revisão é significativamente limitado. No entanto, o estudo destaca considerações importantes sobre a triagem e o risco de desenvolvimento da doença nesse grupo específico de pacientes. Embora a LV seja rara entre os receptores de transplante e frequentemente negligenciada, mesmo em áreas endêmicas, a identificação precoce e o tratamento adequado são cruciais para melhorar os desfechos de sobrevida.

## Conclusão

Esta revisão integrativa avaliou o perfil clínico da LV em receptores e candidatos ao TR, enfatizando a limitação de dados disponíveis para esse grupo. A maioria dos casos de LV exibiu apresentações típicas, embora as formas atípicas tenham representa-do um desafio para o diagnóstico. O envolvimento mucocutâneo concomitante foi associado a taxas de mortalidade mais elevadas. Os testes sorológicos não são realizados rotineiramente para a triagem de infecção assintomática, e os candidatos a TR não apresentaram uma prevalência maior de infecção latente em compa-ração com a população em geral. Embora a função renal possa declinar devido à doença ou ao seu tratamento, a perda do enxerto permanece incomum. Transfusões sanguíneas podem aumentar o risco de infecção, porém a triagem de rotina em bancos de sangue para doadores ou receptores não é recomendada atualmente. São necessários estudos adicionais para uma melhor compreensão do papel da imunossupressão e da profilaxia secundária no manejo da LV.

## Data Availability

Os dados que sustentam as conclusões deste estudo estão disponíveis mediante solicitação, bem como nas tabelas do manuscrito.
